# Hemidiaphragm work in large pleural effusion and its insignificant impact on blood gases: a new insight based on *in silico* study

**DOI:** 10.3389/fphys.2025.1539781

**Published:** 2025-04-11

**Authors:** Tomasz Gólczewski, Anna M. Stecka, Elżbieta M. Grabczak, Marcin Michnikowski, Monika Zielińska-Krawczyk, Rafał Krenke

**Affiliations:** ^1^ Nalecz Institute of Biocybernetics and Biomedical Engineering, Polish Academy of Sciences, Warsaw, Poland; ^2^ Department of Internal Medicine, Pulmonary Diseases and Allergy, Medical University of Warsaw, Warsaw, Poland

**Keywords:** large pleural effusion, therapeutic thoracentesis, hemidiaphragm function, hemidiaphragm inversion, arterial blood gases, virtual patient, in silico study, pendulum breathing

## Abstract

**Objective:**

Computer simulations, enabling observations of variables inaccessible in living patients, provide a powerful approach to studying complex physiological phenomena. This *in silico* study presents the use of a virtual patient to investigate the impact of large pleural effusion (PE) and therapeutic thoracentesis (TT) on hemidiaphragm function and arterial blood gases.

**Methods:**

Inspired by unexpected phenomena observed in living patients undergoing large-volume TT, we formulated four questions regarding this impact. To answer these questions, we simulated right-sided PE in our virtual patient and studied changes in the pleural pressure in the ipsilateral hemithorax (Ppli) and lung volume during the respiratory cycle (exemplified by Ppli-V loops, where V is the volume of both lungs), airflows in the main bronchi, and alveolar O2 (PAO2) and CO2 (PACO2) partial pressures.

**Results:**

Simulations highlighted that: (a) mediastinal compliance critically affects hemidiaphragm work; (b) the 8-shaped Ppli-V loops are associated with hemidiaphragm inversion, where exhalation from the ipsilateral lung occurs during a part of both the inspiratory and expiratory phases, and vice versa; (c) pre-TT PAO2 may be elevated due to reduction of the tidal volume to end-expiratory lung volume ratio; and (d) pre-TT Ppli amplitudes during respiration can exceed post-TT values when mediastinal compliance is high.

**Conclusion:**

Our findings emphasize the significance of mediastinal compliance in pleural effusion physiology and suggest insignificant influence of the ipsilateral hemidiaphragm inverted due to large PE on arterial gas tensions. This study underscores the utility of virtual patient models for elucidating unexpected physiological behaviors and optimizing clinical interventions.

## 1 Introduction

Pleural effusion (PE) may be a sequela of various diseases, including acute and chronic heart failure, pulmonary and pleural infections and malignancies. It is estimated that PE may appear in as many as 20% of all patients with malignant diseases (Gonnelli et al., 2024) and almost half of those with heart failure (Morales-Rull et al., 2018). Thus, PE was a relatively common condition with the annual incidence of 350 per 100,000 in US and, with the increase in the world population and aging in developed countries, its incidence is expected to increase (Piggott et al., 2023; Bodtger and Hallifax, 2020). Therefore, understanding all the phenomena associated with PE and with pleural fluid withdrawal is crucial. While patient-based research remains the primary means to expand knowledge, development of computer modeling can make the use of virtual patients an important supplementary approach.

The direct impact of PE on ventilation is associated with an increase in pleural pressure (P_pl_). PE exerts pressure on the surrounding structures, i.e., the lungs, hemidiaphragm, mediastinum and rib cage (Karkhanis and Joshi, 2012). This results in lung compression with the collapse of ipsilateral lung dependent regions, leading to a decrease in the gas exchange surface area and deformation of other structures. If PE is large, deformation of the ipsilateral hemidiaphragm can be so significant that this hemidiaphragm may become flattened without any meaningful movement during breathing or even inverted with a paradoxical excursion.

Expansion of the rib cage and deformation of the ipsilateral hemidiaphragm contribute to decreased efficiency of inspiratory muscles hindering ventilation (DeBiasi and Feller-Kopman, 2019). Additionally, paradoxical excursion of the inverted hemidiaphragm might result in “pendulum breathing” (Mulvey, 1965). It may be expected that the above and a decrease in gas exchange surface lead to deterioration of blood oxygenation (Thomas et al., 2020). Elevated P_pl_ also exerts pressure on systemic and pulmonary vessels, leading to impaired blood flow (Stecka et al., 2018). Therefore, arterial blood oxygenation is expected to decrease in such patients, particularly in those with large PE, and therapeutic thoracentesis (TT) is expected to significantly increase this oxygenation. However, Taylor et al. reported no statistically significant changes in saturation (Taylor et al., 2021). Zielińska-Krawczyk et al. showed that the partial pressure of oxygen in arterial blood (P_a_O_2_) was greater 1 h after TT than before TT but patients with very large PE exhibited the least pronounced increase (Zielińska-Krawczyk et al., 2022). Surprisingly, the median P_a_O_2_ before TT was slightly higher in patients with very large PE than in the other patients and the median P_a_CO_2_ was slightly lower (Zielińska-Krawczyk et al., 2022). Using a virtual (*in silico*) patient and data from living patients, Stecka et al. demonstrated that various changes in P_a_O_2_ levels, including both a decrease and an increase, are possible (Stecka et al., 2018).

Our medical-engineering team pursues both clinical and theoretical research to evaluate respiratory mechanics during large and very large PE, and its changes during TT. In particular: (a) we utilized changes in transcutaneous partial pressures of O_2_ (P_tc_O_2_) and CO_2_ (P_tc_CO_2_) to evaluate changes in P_a_O_2_ and P_a_CO_2_, (b) to evaluate whether the ipsilateral hemidiaphragm is functionally inverted, i.e., whether P_pl_ in the ipsilateral hemithorax increases during inspiration (which leads to “pendulum breathing”), we analyzed the shape and orientation of the P-V loop (where P is the P_pl_ measured in the ipsilateral hemithorax and V is the current volume of air inhaled to or exhaled from the whole respiratory system): if P at the end of inspiration is greater than at the end of expiration, the loop leans to the left and the ipsilateral hemidiaphragm is treated as functionally inverted.

On the other hand, a large part of our theoretical research bases on virtual experiments with the use of VirRespir (Zieliński et al., 2018), i.e., a versatile virtual cardiopulmonary patient being a set of models working together (Zieliński et al., 2022); however, the respiratory mechanics model was modified to enable simulation of pleural effusion and TT (Gólczewski et al., 2017; Stecka et al., 2018).

Based on seemingly contradictory results presented in the literature and the interesting cases from our database that are presented below, the following four key questions were formulated to be explained by computer simulations:

1. Why can the hemidiaphragm be not functionally inverted despite very large PE?
2. Why are some P-V loops 8-shaped?
3. Why is there no significant and uniform impact on blood oxygenation during pleural fluid withdrawal, despite the notable disturbance in breathing mechanics caused by large PE, particularly in patients with functionally inverted hemidiaphragms?
4. Why may the P_pl_ amplitude in the ipsilateral hemithorax decrease with pleural fluid withdrawal in large PE since it usually increases in the majority of patients (Zielińska-Krawczyk et al., 2018)?

To our knowledge, this study is the first one that analyzes the P-V loop defined above, and, in general, it is one of few works that concern the use of computer modeling to find possible explanations of the phenomena, sometimes surprising, that were observed during TT in living patients, particularly in those with large PE. It should be stressed, however, that computer models enable investigators to formulate only sufficient conditions of the phenomena, which also concerns our study.

## 2 Methods and materials

### 2.1 Virtual patient

Our virtual patient has been used for 20 years in simulations of a broad spectrum of physiological and pathophysiological phenomena associated with ventilation, gas exchange, gas transport, and pulmonary circulation; an educational system was the first application. In essence, our virtual patient comprises models of respiratory system mechanics, pulmonary circulation, gas transfer in bronchi, gas exchange in the lungs and blood gas transport. Additionally, there is a possibility to connect external systemic circulation models of various complexities (Zieliński et al., 2018).

The models collaborate by exchanging calculated variable values, enabling interaction between them. For instance, the gas transfer model utilizes airflow data from the respiratory system mechanics and gas exchange models, while the blood transport model incorporates blood flow data from the pulmonary circulation model. The default values of the respiratory system model parameters were calibrated to match those of an average 50-year-old Polish woman. Parameters in other models were set to correspond to the average adult.

In the standard version of the virtual patient, each lung lobe is subdivided into 16 segments characterized by their volumes and the coordinates of their mass centers in the coordinate system with the origin at the pulmonary trunk. This subdivision enables, for example, the investigation of ventilation-perfusion mismatches in different positions, such as supine, standing or lateral (Zieliński et al., 2018). This division is applied uniformly across all models, including both respiratory system mechanics and pulmonary circulation models. Another division had to be carried out in simulations of PE. The hydrostatic pressure exerted by pleural fluid, which modifies local P_pl_, is crucial, e.g., it influences local airflow and causes the collapse of those lungs parts for which the alveolar pressure is lower than the surrounding local P_pl_. Therefore, the division of lungs into multiple horizontal layers was necessary since, in the sitting position, the hydrostatic pressure progressively increases vertically from the apex to the diaphragm. Initial simulations showed that using more than 100 layers did not significantly alter the simulation outcomes; hence, 100 layers were deemed appropriate.

The model of respiratory system mechanics consists of compartments related to the mouth and trachea, main bronchi, viscoelastic rib cage, elastic mediastinum, two compliant hemidiaphragms, viscoelastic abdomen, and compliance of dead space (separately for each lung) and, for each layer, viscoelastic parenchyma, compliance of the alveolar space (gas compressibility), collapsible bronchi of the middle orders, smallest bronchi and ducts. For mathematical descriptions of the respiratory system and pulmonary circulation compartments that are crucial in this study, please refer to the Supplementary Data.

### 2.2 Study design

The virtual patient was used to propose possible explanations of the variety of blood gas changes and ipsilateral hemidiaphragm work observed during TT in the living patients with large pleural effusion that participated in our clinical study and are presented below. We replicated a scenario of right-sided PE. A pleural fluid volume of only 3 L was simulated, allowing for the maintenance of a small upper part of the ipsilateral lung to analyze possible “pendulum breathing”.

The simulations primarily focused on monitoring P_pl_and both lungs volume changes during the respiratory cycle, exemplified by the P-V loop. The ipsilateral hemidiaphragm work was characterized by the shape and orientation of this loop. To elucidate the variations in observed P-V loops in living patients, we examined the impact of mediastinal, rib cage, abdomen and diaphragmatic compliances. The model coefficient related to one of these factors was initially reduced from its default value in the first series of simulations and then increased in the second series. Simulations performed for such deviations were used to establish (a) parameters crucial for the loop orientation and shape, and (b) airflows in the main bronchi, including the possibility of “pendulum breathing”.

Additionally, we observed alveolar O_2_and CO_2_partial pressures in the main bronchi. The results of these simulations and those concerning changes in pulmonary blood flow during TT presented elsewhere (Stecka et al., 2018) were subsequently used to explain changes in arterial blood gases during this procedure.

As computer simulations enabled us to formulate sufficient conditions, we have not treated our findings as necessary conditions of phenomena observed by us in living patients or reported in the literature, i.e., we admit that the reasons for those phenomena in some patients might be different.

### 2.3 Living patients

#### 2.3.1 Patients

Simulations have been performed to dispel doubts reported in the literature but the main reason for this study was to find possible explanations of interesting cases coming from the database that was created by us within the framework of a previously conducted clinical project related to TT. The protocol of that project, approved by the Institutional Review Board (KB 105/2012), was registered at ClinicalTrials.gov (NCT02192138). The project complied with the standards set out in the Declaration of Helsinki. Medical procedures were conducted with the participation of patients hospitalized in the Department of Internal Medicine, Pulmonary Diseases and Allergy. Patients signed an informed consent to participate in the study beforehand. Details related to the project were presented elsewhere [e.g., (Stecka et al., 2018; Zielińska- Krawczyk et al., 2018; Zielińska-Krawczyk et al., 2022)].

**FIGURE 1:**
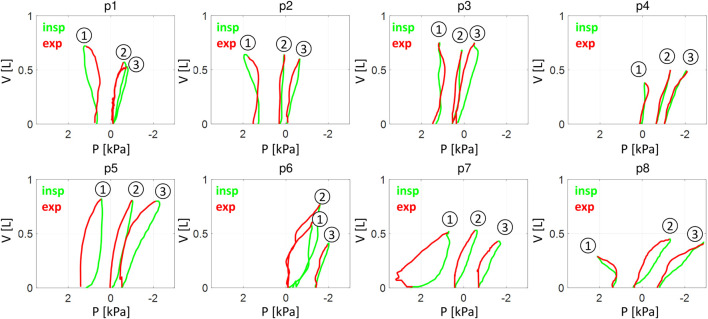
The P-V loops: living patients. P – the pleural pressure in the ipsilateral hemithorax, V – the change in the total lung volume during inspiration (green/light) and expiration (red/dark). 1,2 and 3 - the loops from measurements before pleural fluid withdrawal, after a withdrawal of approximately 1.9 L and after withdrawal of approximately 3.8 L, respectively. p1 … p8 – living patients from Table 1.

The chosen cases concern all patients with the functionally inverted hemidiaphragm (patients p1, p2 and p8 in Figure 1). For comparison, three patients with the greatest volume of withdrawn pleural fluid but with the P-V loops leaned to the right were also included (patients p5, p6 and p7 in Figure 1). Additionally, all patients with vertical P-V loops were included (patients p3 and p4 in Figure 1). Table 1 presents the characteristics of these patients.

**TABLE 1:** Characteristics of living patients.

Patient	Sex	Age [yrs]	Side of PE	Dyspnea change	Vwpf [L]	
p1	M	52	R	4	4.83	I
p2	M	57	L	8	4.80	I
p3	M	58	L	0	4.25	F
p4	M	82	R	4	3.78	F
p5	M	77	R	4	4.65	N
p6	M	91	R	NA	4.00	N
p7	F	82	R	4	4.94	N
p8	F	73	L	NA	2.70	I

M/F – Male/Female; R/L – the right/left side of pleural effusion (PE); V_wpf_ – the total volume of withdrawn pleural fluid; I/F/N functionally inverted/flattened/normal ipsilateral hemidiaphragm (based on P-V loops); dyspnea change caused by TT was quantified using the Borg scale; in the case of p8, TT had to be stopped prematurely due to symptoms (hence, a smaller V_wpf_).

##### 2.3.2 Measurements

The TT procedure under the control of P_pl_ measurements was performed as described elsewhere (Zielińska- Krawczyk et al., 2018; Zielińska-Krawczyk et al., 2022). The following parameters were recorded:

• Instantaneous P_pl_ (digital pleural manometer, IBBE PAS, Warsaw, Poland);
• Airflow at the level of the mouth (modified spirometer, LungTest 1000, MES, Cracow, Poland);
• P_tc_O_2_ and P_tc_CO_2_ (TCM4^TM^ monitoring system, Radiometer, Copenhagen, Denmark) – due to technical problems, these parameters were not measured in the patient p8;
• Heart rate (HR), systolic and diastolic blood pressure (SBP and DBP, respectively) (IGEL ICARD M, IGEL, Gliwice, Poland).

The instantaneous P_pl_ and airflow were recorded at 1-min intervals between the aspiration of the subsequent pleural fluid portions (200 mL up to 1 L and then 100 mL) using the sampling frequency equal to 25 Hz. All signals were synchronized and recorded using Toracemon software (IBBE PAS, Warsaw, Poland). Airflow integration provides the volume of inhaled/exhaled air used for P-V loop creation. Dyspnea was assessed by the 10-point modified Borg scale immediately before TT and after its termination. Minute ventilation (V_E_) was calculated as VT∙RR, where RR was the respiratory rate. Because the stroke volume was not accessible, changes in cardiac output were assessed by changes in the index ∼CO = (SBP–DBP) HR.

##### 2.3.3 Interpreted phenomena

**FIGURE 2:**
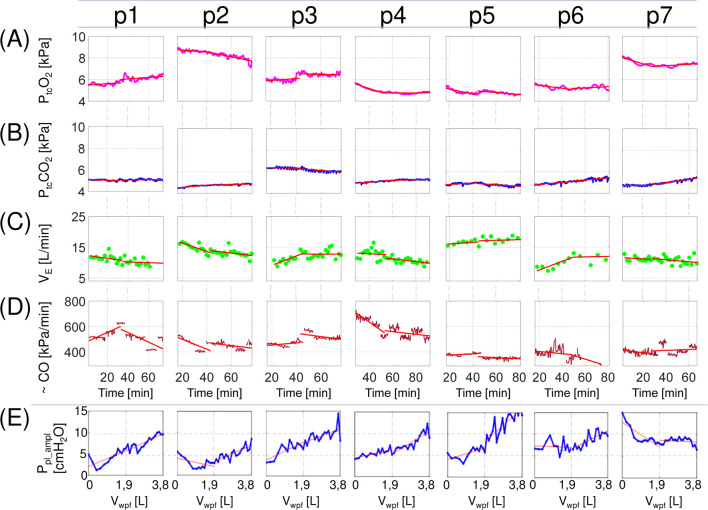
Changes in selected parameters during therapeutic thoracentesis in living patients. P_tc_O_2_ and P_tc_CO_2_- transcutaneous oxygen and carbon dioxide pressure, respectively; V_E_ – minute ventilation; _∼_CO – the product of heart rate and pulse pressure (an estimation of cardiac output); P_pl_ampl_ – the median value of the pleural pressure amplitude form intervals recorded between aspiration of subsequent pleural fluid portions; lines - linear regression of a parameter on time **(A–D)** or volume **(E)** calculated for the first and the second stages of therapeutic thoracentesis. p1 … p7 – living patients from Table A1 (P_tc_O_2_ and P_tc_CO_2_ were not measured in the patient p8 due to technical problems).

The tendencies of changes in P_tc_O_2_, P_tc_CO_2_, V_E_ and ∼CO in real patients are presented in Figures 2A–D. The tendency was characterized quantitatively by means of the slope of the linear regression line (lines in Figure 2; see the Supplementary data for numerical values). In very large PE, withdrawal up to approximately 1.9 L seems to have a different impact on respiratory system work than further withdrawal; e.g., the amplitude of breathing-related P_pl_ changes usually starts to grow steadily after withdrawal of 1.9 L, whereas earlier, it changes in a more diverse way (Figure 2E). Therefore, the slope was calculated separately for volumes <1.9 L (the first TT stage) and for greater volumes (the second stage).

There was no uniform trend in P_tc_O_2_ changes during TT, and different patterns were observed, i.e., no significant changes, a slight increase or decrease, or biphasic alteration (Figure 2A). P_tc_CO_2_ demonstrated overall greater stability and its variations were also not uniform (Figure 2B), which suggest that V_E_ was kept on the level required by the respiratory control centre working mainly on P_a_CO_2_. However, changes in P_tc_O_2_ could not be attributed to variations in V_E_ since correlation between V_E_ and P_tc_O_2_ was close to zero.

The slopes of P_tc_O_2_ and P_tc_CO_2_ exhibited significant correlations only with ∼CO and only during the first stage of TT (r = 0.74 and −0.62, respectively). This, however, may be an artifact, as the ∼CO decrease during this stage is mainly related to the arterial pressure decrease (perhaps due to the alleviation of the initial patient’s stress), which might have an impact on transcutaneous measurements. Indeed, the arterial pressure rise increases the arterial component in microcirculation increasing influence of arterial gases on transcutaneous measurements (hence, the positive correlation for P_tc_O_2_ and the negative correlation for P_tc_CO_2_).

The above and Figure 1 show that:

• TT does not impact blood gases uniformly,
• all P-V loops for the functionally inverted hemidiaphragm were 8-shaped (p1, p2 and p8 before TT in Figure 1),
• despite large or very large PE, the ipsilateral hemidiaphragm may be not functionally inverted (p5, p6 and p7 in Figure 1),
• the P_pl_ amplitude in the ipsilateral hemithorax rapidly decreased in the first stage in some patients (p1, p2 and p7 in Figure 2E) whereas it usually increases with pleural fluid withdrawal (Zielińska- Krawczyk et al., 2018).

**FIGURE 3:**
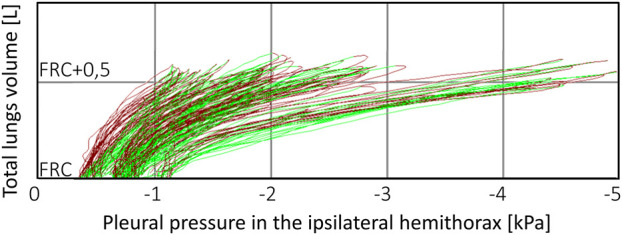
An example of P-V loop changes during therapeutic thoracentesis. Although pleural pressure at functional residual capacity (FRC) moderately fell as pleural fluid was withdrawn, the pleural pressure amplitude increased dramatically, particularly after withdrawal of 1,000 mL of the fluid (which was the reason for therapeutic thoracentesis termination) despite that tidal volume did not change. Presumably, this is the case of patient with the nonexpendable lung and very compliant mediastinum.

Computer simulations were performed to propose possible explanations for these observations. The direct purpose of these simulations was not to explain another interesting observation, i.e., the discrepancy between the changes in P_pl_ at functional residual capacity (FRC) and the changes in the P_pl_ amplitude (Figure 3). However, it should be mentioned because it turned out to be related to the above observations.

##### 2.3.4 Differences between measured and simulated parameters

The actual values of P_a_O_2_ and P_a_CO_2_ in living patients during TT could be obtained using only additional invasive methods being ethically unjustified in the case of severely ill patients. We therefore recorded P_tc_O_2_ and P_tc_CO_2_ throughout the whole procedure whereas simulations concerned P_a_O_2_ and P_a_CO_2_, which is the main discrepancy. Transcutaneous measurement is a well-known technique for monitoring blood gases but some previous studies have not confirmed the significant correlation between P_tc_O_2_ and P_a_O_2_ measured in different subjects (Górska et al., 2015); therefore, P_tc_O_2_ should not be regarded as a perfect substitute for P_a_O_2_ and should not be used in comparisons of results between different patients. We assumed, however, that relative changes in P_tc_O_2_ and P_tc_CO_2_ well imitate relative changes of P_a_O_2_ and P_a_CO_2_ in an individual.

Another discrepancy concerns the lack of direct measurements of CO whereas we controlled CO in the virtual patient; instead, we had to rely on estimations based on SBP, DBP, and HR.

## 3 Results and interpretation of simulations

### 3.1 The first question

#### Why can the hemidiaphragm be not functionally inverted despite very large PE?

**FIGURE 4:**
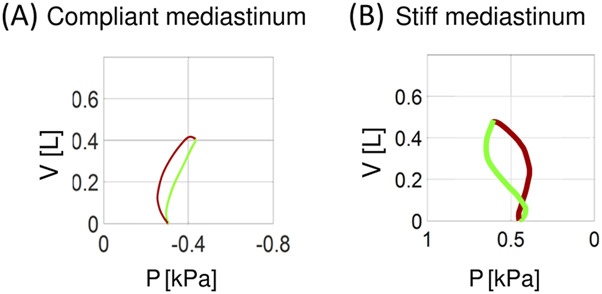
Examples of simulated P-V loops for compliant **(A)** and stiff **(B)** mediastinum. P–the pleural pressure in the ipsilateral hemithorax, V–the change in the total lung volume during inspiration (green) and expiration (red).

As simulations have suggested, the rib cage and abdomen compliances are important for addressing this question. In particular, a stiffer abdomen may protect the ipsilateral hemidiaphragm against significant deformation. However, simulations have shown that the impact of the mediastinum compliance seems to be most significant and much more intriguing. Figure 4 shows examples of the P-V loops from simulations with mediastinum compliance higher (Figure 4A) and lower (Figure 4B) than the default value. If this compliance is high, large PE deforms the mediastinum more than the ipsilateral hemidiaphragm; consequently, this hemidiaphragm may not be inverted, and the P-V loop may physiologically lean to the right. Additionally, even if the ipsilateral hemidiaphragm is “formally” inverted (e.g., the inversion is evident in USG or RTG examination), it need not be functionally inverted in the sense defined above when the mediastinum compliance is high because the contralateral hemidiaphragm affects also the ipsilateral lung through this compliant mediastinum (Figure 4A shows such a case). It might be enhanced in patients with contralateral hemidiaphragm hyperactivity (Fitzgerald et al., 2022). The above might be one possible explanation why the patient p7 had not functionally inverted hemidiaphragm despite very large PE.

### 3.2 The second question

#### Why are some P-V loops 8-shaped?

To our knowledge, 8-shaped P-V loops have not yet been noted or discussed in the literature. Simulations have suggested that the following sequence can explain this shape in patients with a less compliant mediastinum, i.e., with the functionally inverted hemidiaphragm.

##### 3.2.1 The inspiration beginning

**FIGURE 5:**
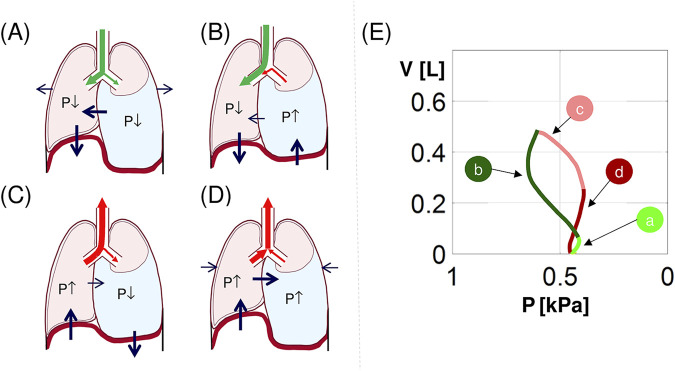
Explanation of the 8-shaped P-V loop. **(a–d)** an illustration of hemidiaphragms, rib cage and mediastinum movements and flows in main bronchi during **(a)** the inspiration beginning, **(b)** the second inspiration phase, **(c)** the expiration beginning, and **(d)** the second expiration phase (green/red arrows–fresh/processed air; black arrows–movement of the structures; P↓/P↑ – decrease/increase in P_pl_ and corresponding change in P_A_); **(e)** the simulated 8-shaped P-V loop from Figure 1B; the letters a-d correspond to **(a–d)** (see the text for details).

**FIGURE 6:**
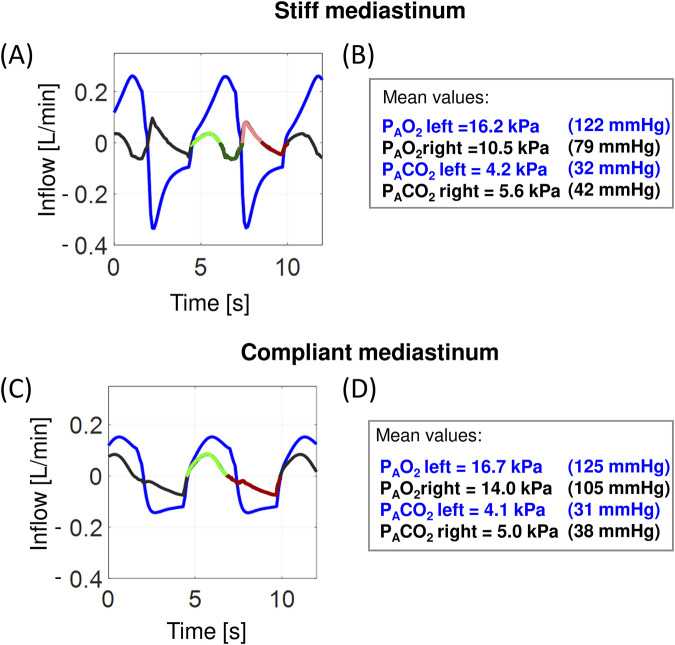
Examples of simulated airflows in the main bronchi and alveolar O_2_ and CO_2_ partial pressures: the virtual patient with PE in the right hemithorax. **(a, c)** inflow to the left bronchus (blue) and the right bronchus (black and in one respiratory cycle: light/dark green–inflow/outflow during inspiration, light/dark red–inflow/outflow during expiration; these colors correspond to the colors in Figure 2E); **(b, d)** the mean values of the alveolar oxygen partial pressure (P_A_O_2_) and the alveolar carbon dioxide partial pressure (P_A_CO_2_), respectively.

Before inspiration begins, the contralateral hemidiaphragm, stretched by the negative P_pl_ in the contralateral hemithorax, is longer than the ipsilateral hemidiaphragm (supported by abdominal organs). Therefore, according to the force‒length relationship of the muscles, initial diaphragm contraction means mainly contraction of the contralateral hemidiaphragm. A decrease in the P_pl_ in the contralateral hemithorax propagates through the mediastinum to the ipsilateral hemithorax. Additionally, the activity of rib cage muscles decreases P_pl_ in both hemithoraxes. The above causes:

(a) flow of fresh air into both lungs (Figure 5A and the light green part in Figure 6A) like in the case of not inverted hemidiaphragm (the light green part in Figure 6C),
(b) the part of the P-V loop that is directed to the right, i.e., to the smaller P (the light green line in Figure 5E).

##### 3.2.2 The rest of inspiration

Further diaphragm contraction causes upwards movement of the inverted ipsilateral hemidiaphragm, and this activity predominates over the influence of the contralateral hemidiaphragm that is only slightly propagated through the stiffer mediastinum. This increases P_pl_ and, consequently:

(a) processed air flows out from the ipsilateral lung and mixes with fresh air still flowing into the contralateral lung (Figure 5B and the dark green line in Figure 6A) unlike the case of not inverted hemidiaphragm (the rest of the light green part in Figure 6C),
(b) the second part of the P-V loop is directed to the left, i.e., to the greater P (the dark green line in Figure 5E).

##### 3.2.3 The beginning of expiration

The inverted hemidiaphragm starts to relax, i.e., it moves down, which decreases P_pl_. This causes that:

(a) processed air exhaled from the contralateral lung flows into the ipsilateral lung (Figure 5C and the light red line in Figure 6A),
(b) P_pl_ in the ipsilateral hemithorax decreases with decreasing V (the light red line in Figure 5E).

##### 3.2.4 The rest of expiration

Further influence of the inverted hemidiaphragm is insignificant in comparison with the influence of the contralateral hemidiaphragm; thus, the airflows in both lungs are similar, which gives:

(a) exhalation of processed air from both lungs (Figure 5D and the dark red line in Figure 6A),
(b) P_pl_ in the ipsilateral hemithorax increases with decreasing V (the dark red line in Figure 5E).

The above explains the 8-shaped loop. Note that although the ipsilateral hemidiaphragms of patients p3 and p4 were initially assumed to be not inverted, their 8-shaped P-V loops suggested that the hemidiaphragm was slightly inverted also in these patients since the 8-shape requires the above.

### 3.3 The third question

#### Why is there no significant and uniform impact on blood oxygenation during pleural fluid withdrawal, despite the notable disturbance in breathing mechanics caused by large PE, particularly in patients with functionally inverted hemidiaphragm?

It might be expected that large or very large PE, which significantly disturbs ventilation, should worsen blood oxygenation; consequently, pleural fluid withdrawal should be associated with significant improvement in blood oxygenation. This improvement, however, was not observed in all living patients (Stecka et al., 2018; Taylor et al., 2021), particularly in those presented above. As the virtual patient is fully observable, we could identify the three following phenomena, which enabled us to answer this problem.

First, increased P_pl_ in the ipsilateral hemithorax significantly suppresses blood flow [e.g., see corresponding simulations presented elsewhere (Stecka et al., 2018)]; thus, almost all the blood flows through the ventilated contralateral lung. As pleural fluid is withdrawn, P_pl_ in the ipsilateral hemithorax decreases, and blood flows also through the ipsilateral lung regardless of whether the collapsed lung parts are recruited and ventilated; consequently, if these parts are not recruited, TT may temporarily worsen oxygenation, not improve, due to the pulmonary shunt.

Second, since, before TT, the majority of blood flows through the contralateral lung, the rate of gas exchange in this lung is greater, which causes P_A_O_2_ during expiration to be lower than normal. As pleural fluid is withdrawn, blood may flow through the ipsilateral lung, and this lung may also participate in gas exchange to an increasing extent; consequently, P_A_O_2_ may not fall as much as before TT giving more stable gas exchange.

Third, fresh air is mixed during inspiration with the processed air that remains in the lungs and bronchi after preceding expiration, and the volume of this processed air is equal to the sum of the FRC and anatomical dead space (V_D_). The V_T_/(FRC+V_D_) ratio, were V_T_ is the tidal volume, determines the composition of the mixture of fresh and processed air (i.e., P_A_O_2_ and P_A_CO_2_) during inspiration; for example, a typical P_A_O_2_ value is lower by approximately 8 kPa (60 mmHg) than the partial pressure of O_2_ in the atmospheric air. If the ipsilateral lung is collapsed and V_T_ remains at a desired level (as in our simulations), this ratio may even be two times greater because FRC+V_D_ concerns only the contralateral lung; hence, the mean P_A_O_2_ in this lung can be higher than normal, and P_A_CO_2_ can be lower (the values for the left lung in Figures 6B, D). As pleural fluid is withdrawn, the ratio may decrease due to the requirement of collapsed parts of the ipsilateral lung; consequently, P_A_O_2_ in the contralateral lung during inspiration also decreases. This may explain the intriguing observation that blood oxygenation improvement after TT is smallest in patients with large PE (Zielińska-Krawczyk et al., 2022).

### 3.4 The fourth question

#### Why may the P_pl_ amplitude in the ipsilateral hemithorax decrease with pleural fluid withdrawal in large PE since it usually increases in the majority of patients?

In all the cases analyzed by Zielińska et al., the P_pl_ amplitude increased with pleural fluid withdrawal at the first stage of TT (Zielińska-Krawczyk et al., 2018), which seems to be understandable since the withdrawal improves the ipsilateral hemidiaphragm work. However, in some of the cases presented above, the amplitude initially decreases. This decrease may be easily explained in patients with the functionally inverted hemidiaphragm. Indeed, during TT, the P-V loop in such patients leans to the left, then becomes vertical and, finally, leans to the right (e.g., see the loops for the patients p1 and p2). Since the P_pl_ variations are smallest when the loop is vertical, the P_pl_ amplitude is initially higher, then decreases according to the loop rotation, and finally increases.

However, the decrease in the P_pl_ amplitude in the patient p7 was surprising since her hemidiaphragm worked normally despite very large PE (probably due to high mediastinum compliance). Simulations suggested the following possible explanation. Since very large PE (at least 5 L in this patient) leads to collapse of the whole or a considerable part of the ipsilateral lung, the total lungs compliance significantly decreases because a significantly smaller part of the lungs is involved in ventilation. Moreover, the same V_T_ requires a greater expansion of the contralateral lung to the volume for which compliance of the ventilated lung is small due to lung compliance nonlinearity (e.g., see the Supplementary file for the mathematical equation that describes this nonlinearity in the virtual patient). As a consequence, the amplitude of breathing-related P_pl_ changes in the contralateral hemithorax must be much greater than normal to preserve the required V_T_ (this can be interpreted as compensatory hyperactivity of the contralateral hemidiaphragm (Fitzgerald et al., 2022)). If the mediastinum is very compliant, then these large P_pl_ changes may easily propagate to the ipsilateral hemithorax, leading to the P-V loop that is leaned to the right but with great P variation. When the total lungs compliance increases due to the recruitment of collapsed parts, the amplitude decreases. Note that the P_pl_ amplitude did not change in the second stage of TT (Figure 2E), which additionally confirms the recruitment.

## 4 Discussion

Knowledge of pleural space physiology is not complete (Hu et al., 2020). This is due in part to difficulties in measurement of key physiological parameters. In particular, studies of ipsilateral hemidiaphragm activity and its influence on airflows, P_A_O_2_ and P_A_CO_2_ in individual bronchi are impossible in living patients because measurements of these parameters are unfeasible. The same concerns analyses of mediastinum behaviour during TT. Therefore, experiments *in silico* seem to be the only way to gain understanding of pleural space physiology. To our knowledge, this study is the first one that presents simulations of diaphragm and mediastinum functions in large PE. As our virtual patient made it possible to observe factors inaccessible in the case of living patients, it enabled us to propose sufficient conditions for the interesting phenomena that are presented above.

Our simulations primarily enable insight into influence of the mediastinum on the breathing mechanics in large PE; the mediastinal compliance appeared as a key parameter. To our knowledge, this influence has not been discussed yet despite that a number of authors recognized and discussed mediastinal shift towards the contralateral hemithorax [e.g. (Muruganandan et al., 2020; Cho et al., 2022; Polski et al., 2023)] or even towards the ipsilateral hemithorax when PE is accompanied by bronchial obstruction leading to ipsilateral lung atelectasis (Khajotia, 2012).

First of all, the mediastinum can transmit or not the P_pl_ changes that are related to breathing, which depends on the value of its compliance. If the mediastinum is compliant then decrease of P_pl_ during inspiration in the contralateral hemithorax is transmitted to the ipsilateral hemithorax, which can balance paradoxical ipsilateral hemidiaphragm movement giving normal P-V loop (Figure 4A) despite that RTG or USG shows ipsilateral hemidiaphragm inversion. If, however, mediastinum is stiff, and thus it separates both hemithoraxes, then the ipsilateral hemidiaphragm may be both anatomically and functionally inverted, which is a worse case (Figure 4B). For example, two results (i.e., the P-V loop leaned to the right and the great P_pl_ amplitude before TT; Figure 1) suggest that the patient p7 has a high mediastinal compliance; therefore, although she had the greatest PE volume despite the smallest height, she reported less severe dyspnea before TT than most others (Table 1). As thoracic radiation therapy may lead to fibrosis also involving the mediastinum making it stiff (Lin et al., 2004; Zhao et al., 2017), we hypothesize that if a patient suffers from malignant PE and is treated with this therapy, he/she may be in a worse condition before TT than others.

Mediastinum compliance can also help explain other unresolved issues. For example, there is controversy about whether pleural manometry is useful to protect against complications caused by excessive P_pl_ fall during large-volume TT or not (Hassaballa et al., 2023; Lentz et al., 2019). On the other hand, Chopra et al. showed discrepancy in the detection of incomplete lung expansion between post-TT pleural manometry and radiographic examination (Chopra et al., 2020). In our opinion, these controversy and discrepancy would disappear if both P_pl_ and the P_pl_ amplitude were analyzed. In particular, the role of the mediastinum cannot be adequately assessed without such an analysis. Unfortunately, even if P_pl_ is monitored during TT by other authors, the P_pl_ amplitude seems to be completely absent in studies concerning problems related to PE and effects of TT [e.g. (Hu et al., 2020; Hassaballa et al., 2023)]. For example, if the mediastinum is very compliant, then even if the ipsilateral lung is not expanded completely, P_pl_ at FRC may not fall extensively because the contralateral lung takes the place of withdrawn pleural fluid. On the other hand, if the ipsilateral lung is not expanded completely, the total lung compliance is still decreased and thus the P_pl_ amplitude has to be greater to provide the required tidal volume. Figure 3 presents the P-V loops obtained for a patient with such a moderate fall of P_pl_ at FRC and the very great increase in the P_pl_ amplitude (TT was terminated in this patient just due to symptoms caused by this amplitude).

Simulations have suggested the following sequence explaining small influence of large PE on arterial blood gases. Before TT, almost all the blood flows through the ventilated (contralateral) lung with P_A_O_2_ higher than normal during inspiration due to the big value of the ratio VT/(FRC+VD) and lower than normal during expiration due to more intensive gas exchange in this lung. Consequently, before TT, the average P_a_O_2_ in mixed blood in the systemic arterial system may be either lower or higher or within the physiological range despite large PE. Additionally during and after TT the pulmonary shunt may be present or not. As these three phenomena have opposite influences on blood oxygenation, the final effect related to pleural fluid withdrawal depends on which phenomenon suppresses the others; hence, there is no uniform reaction to the withdrawal (Stecka et al., 2018; Taylor et al., 2021; Muruganandan et al., 2020).

Although the problem related to possible pendulum breathing in patients with hemidiaphragm inversion and its adverse influence on P_A_O_2_ and gas exchange in lungs was raised decades ago [e.g. (Mulvey, 1965)], it has not yet been finally resolved as neither airflows in bronchi nor partial pressures in particular regions of lung are observable in living patients. Therefore, only conjectural consideration has been possible [e.g. (Thomas et al., 2020)]. Our simulation showed that “pendulum breathing” may be present only at the end of inspiration and beginning of expiration. This is supported by the 8-shaped form of the P-V loop observed in both the virtual patient and living ones. And since the “pendulum breathing” may be present during only a part of inspiration, the mean P_A_O_2_ in the contralateral lung need not be low. Thus, in conclusion, whether pendulum breathing exists or not, large PE has an insignificant influence on blood gases.

Finally, note that the oxygenation did change significantly during TT in neither the virtual patient nor living ones, whereas dyspnea substantially decreased after TT (Table 1); therefore, we support opinion that factors other than arterial blood gases are mainly responsible for the dyspnea frequently experienced by patients with large PE (Muruganandan et al., 2020; Skaarup et al., 2020; Fjaellegaard et al., 2024). However, since dyspnea is a subjective symptom, it cannot be simulated by computer models. However, our work shows that computer simulations of objective physiological phenomena can be very helpful in both research and education (Tomalak et al., 2008; Zieliński et al., 2022).

### 4.1 Study limitation

The lack of a more precise representation of components in the virtual patient’s abdomen is a very important limitation. In particular, the liver as such is not modeled, whereas it supports the right hemidiaphragm, which is postulated to be the reason for less frequent inversion of this hemidiaphragm (Mulvey, 1965), which is also our case (Table 1). The influence of the liver was simulated indirectly only as a decrease in the abdominal compliance. Nevertheless, our simulations seem to be reliable since the P-V loops from the simulations match well with the loops observed in patients, even in those with the 8-shaped loop (e.g., compare Figure 4B and the loop for patient p1).

## 5 Conclusion

Computer simulations offered a new insight into mechanisms of respiration in large PE. It enabled to propose possible explanations of such interesting observations related to TT as the slight change in blood oxygenation in patients with large PE compared with patients with less severe PE, the necessity of hyperactivity of the contralateral hemidiaphragm, the peculiar 8-shaped form of the P-V loop, and insignificant influence of ‘pendulum breathing’ on arterial gas tensions in patients with hemidiaphragm inversion.

## Data Availability

The raw data supporting the conclusions of this article will be made available by the authors, without undue reservation.
