# Supplementary material for: Hemidiaphragm work in large pleural effusion and its insignificant impact on blood gases: a new insight based on in silico study
Source: Front Physiol. 2025 Apr 11;16:1539781. doi: 10.3389/fphys.2025.1539781 (PMC12021632; doi:10.3389/fphys.2025.1539781)
Supplement: Supplementary file 1 [file DataSheet1.pdf]

Supplementary Material

Tendencies of changes in selected parameters during TT.

	$P_{tc}O_2$		$P_{tc}CO_2$		V_E		~ CO	
	Slope [kPa/min] ([mmHg/min])		Slope [kPa/min] ([mmHg/min])		Slope [L/min^2]		Slope [kPa/min^2] ([mmHg/min^2])	
	I	II	I	II	I	II	I	II
p1	0.006 (0.045)	0.006 (0.045)	0.000 (0.000)	0.000 (0.000)	-0.0480	-0.0120	3.67 (27.5)	-3.77 (-28.3)
p2	-0.006 (-0.045)	-0.024 (-0.180)	0.012 (0.090)	0.006 (0.045)	-0.1380	-0.0480	-3.87 (29.0)	-1.13 (-8.5)
p3	0.006 (0.045)	-0.006 (-0.045)	0.000 (0.000)	-0.012 (-0.090)	0.1560	0.0000	0.84 (6.3)	-1.29 (-9.7)
p4	-0.036 (-0.270)	0.000 (0.000)	0.006 (0.045)	0.000 (0.000)	-0.0180	-0.0420	-6.42 (-48.2)	-1.05 (7.9)
p5	-0.024 (-0.180)	-0.012 (-0.090)	0.006 (0.045)	-0.006 (-0.045)	0.0240	0.0120	0.51 (3.8)	-0.46 (-3.4)
p6	-0.012 (-0.090)	0.006 (0.045)	0.006 (0.045)	0.012 (0.090)	0.1440	0.0060	-0.85 (-6.4)	-2.83 (-21.8)
p7	-0.030 (-0.225)	0.006 (0.045)	0.000 (0.000)	0.018 (0.135)	-0.0240	-0.0300	-0.90 (-6.8)	0.37 (2.7)

p1 … p7 – living patients from Table 1. Slope – the slope of the linear regression of a parameter on time. I - the first stage of TT: fluid withdrawal up to 1.9 L; II - the second stage; $P_{tc}O2$ - transcutaneous oxygen pressure; $P_{tc}CO2$ - transcutaneous carbon dioxide pressure; V_E – minute ventilation; ~CO – estimation of cardiac output changes with changes in the product of the pulse pressure and heart rate.

* * *

Chosen compartments of the virtual patient

The most significant influence of pleural effusion on ventilation and blood oxygenation is related to the impact of the hydrostatic pressure exerted by pleural fluid on the lungs, bronchi and pulmonary vessels: if this pressure significantly increases the local pleural pressure (P_{pl}) in dependent regions, it may cause collapse of both the alveoli, bronchi and pulmonary vessels. Therefore, the equations that describe the compliant properties of these elements are crucial and presented below (more details related to the use of the virtual patient in simulations of pleural effusion and therapeutic thoracentesis can be find elsewhere (Gólczewski et al., 2017; Stecka et al., 2018)).

A <u>particular lung part</u> (e.g., the layer when pleural effusion is simulated) is described by the following equation (Gólczewski, 2008; Gólczewski, 2010; Zieliński et al., 2022):

$$Ptps(V) = \frac{\tan\left(\frac{\pi}{2} \cdot \frac{V_A - V_{A0}}{Size}\right)}{(\rho / \rho_0) \cdot Compl}$$

where:

V_A - the current volume of this part;

Ptps - the static transpulmonary pressure (the recoil pressure) equal to the difference between P_A and local P_{pl} if the part tissue does not move;

Size - a parameter related to the part size;

V_{A0} - the lung volume when the recoil pressure is equal to zero (i.e., the atmospheric pressure);

Compl - a parameter characterizing compliant properties of the lung parenchyma; it can be interpreted as the specific compliance at $V_A=V_{A0}$;

ρ_0 - the normal, physiological surface concentration of the surfactant;

ρ - the current surfactant surface concentration (if for any reason $\rho>\rho_0$, then the value of ρ/ρ_0 is assumed to be equal to 1).

Note that if for a part $P_A<P_{pl}$ (i.e., Ptps<0), then V_A has the minimal possible volume; thus, this part is collapsed.

Bronchi of the middle order in a part are simulated as one collapsible tube and described with the following equation derived elsewhere (Gólczewski and Darowski, 2006):

$$Q = \frac{Pb - P_X - b \cdot \arctan\left(b \cdot \frac{Pb - P_X}{b^2 + (Pb - Pp) \cdot (P_X - Pp)}\right)}{k}$$

where:

Q - the airflow through this tube to/from the part,

Pb - the pressure inside the tube at the proximal end, i.e., in the left or right main bronchus (depending on which lung is considered);

P_X - the pressure at the distal end;

Pp - the local P_{pl};

k - the parameter that characterizes the resistive properties of the bronchi and corresponds to the resistance of airway in this part for a very large lung volume;

b - a parameter characterizing the elastic properties of the bronchi.

It can be shown that if Pp>Px, then $Q = (-Ptp(V) + b \times \arctan((Ptp(V)/b)/k$, and thus, the airflow depends only on the part volume, which is the foundation of the forced spirometry[4]. Certainly, if V=0 due to collapse of this part, there is no flow.

Smallest bronchi and ducts of a part are also simulated by one tube. Like alveoli, they are components of the parenchyma; therefore, their current lumens, and thus also their resistance, depend on the current lung volume, in general (Gólczewski, 2010; Gólczewski and Darowski, 2006). If a part is collapsed, the mean resistance of the tube is almost infinite (the resistance is high but not infinite at the proximal end connected with the tube simulating bronchi of the middle order). If the local P_{pl} around the collapsed part falls below the

atmospheric pressure, some airflows exist at the proximal part, which increases the lumen of the other tube parts and thus decreases the resistance enabling the flow of air into the collapsed alveoli. The value of this airflow determines the rate of part recruitment.

The relationship between the **ribcage** and the **mediastinum** is described by the following equations (Gólczewski, 2010):

Pp_L-Pp_R =Pm; Pm=(V_L-V_R)/Cm; Pw=(Pp_L+Pp_R)/2

where Pp_L and Pp_R are the mean P_{pl} in the left and right hemithoraxes, respectively.If *Pm* is not equal to zero due to the difference between the volumes of the left and right hemithoraxes (V_L and V_R, respectively), it acts on the mediastinum having compliance characterized by *Cm*. As has been shown by several authors (e.g., (De Groote et al., 2004)), the chest wall expands symmetrically, even when these two volumes are significantly unequal. Therefore, we assumed that the pressure *Pw* acting on the ribcage is the mean of Pp_L and Pp_R.

Three kinds of equations describe **pulmonary vessels** depending on their size (for details and derivations, see the Supplement for (Stecka et al., 2018)).

Greatest vessels are described with the following equation:

$$P_{tm} = \frac{E_0}{a} \cdot (e^{a(V-V_0)} - 1)$$

where: P_{tm} – the transmural pressure, V – the vessel volume, V_0 is V for P_{tm}=0, E_0– the elastance of the unstressed vessel, *a* – a coefficient.

Smaller vessels are described with the following linear equation:

$$P_{tm} = \frac{V-V_0/\alpha}{c_1/\alpha} = \frac{V \cdot \alpha - V_0}{c_1}$$

where α and C_1 characterize the ability to cause hypoxic vasoconstriction and the vessel compliance, respectively.

The above vessels are compliant but their resistance is not very significant; therefore, we have assumed that there is no significant drop in the transmural pressure along these vessel, determining its volume. This enabled us to directly connect the vessel volume and its resistance according to the Hagen–Poiseuille law. **Capillaries** are both significantly compliant and significantly resistive; thus, a partial differential equation had to be used for their mathematical description (Stecka et al., 2018). We have chosen a form of the differential equation, which enabled to solve it manually. The following solution is used in the virtual patient:

$$R_{cap} = \frac{P_{pro} - P_{dis}}{Q_{blood}} = \left(\frac{1}{g_{cap} \cdot (P_{pro}^2 + P_{pro} \cdot P_{dis} + P_{dis}^2)} \right)$$

where R_{cap} is the Ohm resistance, i.e., the ratio of the difference between the pressures at the proximal and distal ends (P_{pro} and P_{dis}, respectively), and the blood flow Q_{blood} (see the Supplement for (Stecka et al., 2018) for the meaning of the coefficient g_{cap}).

Intercostal muscle activity during breathing is simulated by means of a pressure source, whereas **hemidiaphragm** activity is simulated by changes in the volume between the unstressed hemidiaphragm and the horizontal plane through the diaphragm origin (Gólczewski et al., 2017).

References

De Groote, A., Van Muylem, A., Scillia, P., Cheron, G., Verleden, G., Paiva, M., et al. (2004). Ventilation asymmetry after transplantation for emphysema. Am J Respir Crit Care Med, 170(11), 1233–1238. doi: 10.1164/rccm.200403-323OC

Gólczewski, T. (2010). Models. In: Virtual respiratory system in research and education - principles and applications. IBBE PAS Works No. 74. Warsaw, pp. 23–64.

Gólczewski, T. (2008). e-spirometry™ - an interactive system for e-learning how to interpret the results of spirometry. Available at: http://www.virtual-spirometry.eu/en/index.html (Accessed December 4, 2024).

Gólczewski, T. and Darowski, M. (2006). Virtual respiratory system for education and research: Simulation of expiratory flow limitation for spirometry. Int J Artif Organs, 29(10), 961–972. doi: 10.1177/039139880602901007

Gólczewski, T., Stecka, A.M., Grabczak, E.M., Zieliński, K., Michnikowski, M. and Korczyński, P., et al. (2017). The use of a virtual patient to follow pleural pressure changes associated with therapeutic thoracentesis. Int J Artif Organs, 40(12), 690–695. doi: 10.5301/ijao.5000636

Stecka, A.M., Gólczewski, T., Grabczak, E.M., Zieliński, K., Michnikowski, M. and Zielińska-Krawczyk, M., et al. (2018). The use of a virtual patient to follow changes in arterial blood gases associated with therapeutic thoracentesis. Int J Artif Organs, 41(11), 690–697. doi: 10.1177/0391398818793354

Zieliński, K., Gólczewski, T., Kozarski, M. and Darowski, M. (2022). Virtual and artificial cardiorespiratory patients in medicine and biomedical engineering. Membranes, 12(6), 548. doi: 10.3390/membranes12060548
